# Long-Lived Antibody and B Cell Memory Responses to the Human Malaria Parasites, *Plasmodium falciparum* and *Plasmodium vivax*

**DOI:** 10.1371/journal.ppat.1000770

**Published:** 2010-02-19

**Authors:** Jiraprapa Wipasa, Chaisuree Suphavilai, Lucy C. Okell, Jackie Cook, Patrick H. Corran, Kanitta Thaikla, Witaya Liewsaree, Eleanor M. Riley, Julius Clemence R. Hafalla

**Affiliations:** 1 Research Institute for Health Sciences, Chiang Mai University, Chiang Mai, Thailand; 2 Department of Infectious and Tropical Diseases, London School of Hygiene and Tropical Medicine, London, United Kingdom; 3 Vector Borne Disease Section, Office of Disease Prevention and Control, Chiang Mai, Thailand; Case Western Reserve University, United States of America

## Abstract

Antibodies constitute a critical component of the naturally acquired immunity that develops following frequent exposure to malaria. However, specific antibody titres have been reported to decline rapidly in the absence of reinfection, supporting the widely perceived notion that malaria infections fail to induce durable immunological memory responses. Currently, direct evidence for the presence or absence of immune memory to malaria is limited. In this study, we analysed the longevity of both antibody and B cell memory responses to malaria antigens among individuals who were living in an area of extremely low malaria transmission in northern Thailand, and who were known either to be malaria naïve or to have had a documented clinical attack of *P. falciparum* and/or *P. vivax* in the past 6 years. We found that exposure to malaria results in the generation of relatively avid antigen-specific antibodies and the establishment of populations of antigen-specific memory B cells in a significant proportion of malaria-exposed individuals. Both antibody and memory B cell responses to malaria antigens were stably maintained over time in the absence of reinfection. In a number of cases where antigen-specific antibodies were not detected in plasma, stable frequencies of antigen-specific memory B cells were nonetheless observed, suggesting that circulating memory B cells may be maintained independently of long-lived plasma cells. We conclude that infrequent malaria infections are capable of inducing long-lived antibody and memory B cell responses.

## Author Summary

It is widely perceived that immunity to malaria is short-lived, rendering people susceptible to repeated malaria infections. However, there have been very few studies on “memory” responses, how the human immune system recognizes previously encountered malaria parasites. In particular, very little is known about the durability of malaria-specific B cells and antibodies. The aim of this study was to investigate the induction and maintenance of B cell memory responses to malaria parasites in a region of Thailand where people become infected with malaria, but where the levels of malaria transmission are so low that repeated infection is uncommon. From hospital records we were able to identify people who either had been infected with malaria over the past 6 years and/or had never been infected. Blood samples were collected on four separate occasions over a period of one year and analysed by microscopy and PCR for presence of malaria parasites and by ELISA and ELISPOT for anti malarial antibodies and malaria-specific memory B cells. We found that, in a significant proportion of individuals, malaria infection results in the generation of antibodies and the establishment of populations of memory B cells against malaria parasites, which were very stably maintained over time despite the lack of any evidence of malaria reinfection. Contrary to the widely held idea that memory to malaria is suboptimally induced, our data demonstrate that B cell responses to malaria can be maintained for many years after a malaria infection and indicate that there is no inherent reason why malaria vaccines should not also induce long-lasting protection against malaria.

## Introduction

Malaria, a parasitic disease of humans caused predominantly by two species of *Plasmodium*, *P. falciparum and P. vivax*, remains an important cause of mortality and morbidity in many parts of the world. Development of a vaccine against malaria has proven challenging due to the complex nature of the parasite and to the difficulty in correlating naturally-acquired immune responses with clinical immunity. While immunity against some of the severe clinical symptoms may be achieved quite rapidly, following perhaps as few as one or two infections [1], immune effector mechanisms capable of controlling parasite growth develop only after repeated infections over a number of years. Even with repeated infections, protective immunity to malaria is not complete, and asymptomatic infections may exist throughout life. Understanding the causes of this continuing susceptibility to infection and, in particular, understanding the development and maintenance of immunological memory, is essential for rational development of malaria vaccines.

Antibodies are a crucial component of naturally acquired protective immunity against blood stage malaria with roles that may include inhibition of merozoite invasion into new red blood cells (RBCs), blocking cytoadherence of infected RBCs (iRBCs) to endothelial cells, and enhancing phagocytic activity of monocytes and macrophages (reviewed in [2],[3]). It is widely believed that periodic reinfection is required to maintain acquired immunity to malaria and that antimalarial antibodies are short-lived in the absence of reinfection (reviewed in [4]); implying that B cell memory to malaria may be defective or suboptimal. However, the development and persistence of B cell memory following malaria infection has long been a matter of debate (reviewed in [5]). Some studies in animal models have shown that memory B cells do develop and are maintained normally after malaria infection [6],[7]; whereas others have found that malaria infection interferes with the development of memory B cells and long-lived plasma cells [8],[9]. In humans, several studies have demonstrated stable antibody responses to malaria antigens [10],[11],[12], however, short-lived antibody responses have also been observed [13],[14], especially in young children [10],[15]. To date, very few studies have examined the induction and maintenance of malaria-specific memory B cells in humans. Dorfman *et al* [14] were frequently unable to detect circulating malaria-specific B cells in antibody seropositive children, but it is unclear whether this reflects an absence of such cells or a lack of sensitivity in the assays used to detect them. Conversely, Asito et al [16] observed an increase in both the total CD38^+^IgD^−^ memory B cell population and the transitional CD10^+^CD19^+^ B cell population, following an episode of acute malaria in African children but this study lacked any analysis of the specificity of B cell responses as well as any long term follow up to ascertain the duration of the response.

The aim of this study was to investigate the longevity of the human B cell memory response to malaria in individuals with one or more known malaria infections. To do this, we identified individuals living in an area of very low malaria endemicity in Northern Thailand who were either malaria naïve or who had had recorded (and parasitologically confirmed) clinical episodes of *P. falciparum* or *P vivax* infection some years previously and characterised the antibody and memory B cell response to a variety of discrete *P. falciparum* and *P. vivax* antigens under conditions of infrequent re-exposure/boosting of the immune response.

## Results

### Characteristics of the study subjects at recruitment

Malaria-specific humoral immune responses of 93, HIV negative Thai adults were studied (Table 1). Individuals were assigned to one of three groups according to their place of residence and their prior malaria history. Subjects from Chiang Mai were designated “City Naïve” (n = 17). Subjects from Muang Na (Chiang Dao) were designated “Rural with no clinical malaria episode (Rural 1; n = 30)” if they reported no prior episodes of malaria infection and/or if no record of malaria infection was found in the past 6 years.

**Table 1:** Characteristics of study subjects at recruitment.

	City	Rural 1	Rural 2
**Total (at recruitment) - no.**	17	31	46
**Sex - no. (%)**			
Male	8 (47.1)	11 (35.5)	25 (54.3)
Female	9 (53.9)	20 (64.5)	21 (46.7)
**Age - yr.**			
Mean ± SD	34.7±8.1	32.6±8.5	33.7±7.3
Range	23–46	19–46	19–48
**Recorded malaria episodes** [a] **- no. (%)**			
*P. falciparum* only			21 (45.7)
*P. vivax* only			14 (30.4)
Both *P. falciparum* and *P. vivax*			6 (13.0)
Unknown			5 (10.9)
**Frequency of recorded malaria episodes** [a ^**,**^ b] ** - no.**			
Mean *P. falciparum* ± SD			1.25±0.56
Range *P. falciparum*			1–3
Mean *P. vivax* ± SD			1.10±0.26
Range *P. vivax*			1–2
**Time since last malaria episode** [b] **- mo.**			
Mean *P. falciparum* ± SD			21.2±12.9
Range *P. falciparum*			4–58
Mean *P. vivax* ± SD			20.6±10.1
Range *P. vivax*			7–39

[a] Malaria episodes that were within 45 days apart were considered as a single episode.

[b] Malaria episodes based on records from the Office of Vector Borne Disease Control, Department of Communicable Diseases Control, the Ministry of Public Health, Thailand.

Muang Na residents who had had one or more fully documented episodes of infection with *P. falciparum*, *P. vivax* or both parasite species, as well as those who recalled a previous infection and were seropositive to *P. falciparum* schizont extract (PfSE) but for whom hospital records could not be found, were designated as “previously malaria infected” (Rural 2; n = 46). In this group, 21 subjects (45.7%) reported at least one episode of infection with *P. falciparum*, 14 (30.4%) reported at least one episode of infection with *P. vivax* and 6 (13.0%) reported infection with both species in the past 6 years. The frequency of malaria infections within the six years prior to recruitment varied from 1–3 episodes (mean 1.25±0.56 episodes for *P. falciparum* and 1.10±0.26 for *P. vivax*). Five Rural 2 subjects (10.9%) were strongly seropositive to PfSE and recalled prior malaria episodes, but no documentary evidence of these malaria episodes was found. The time since last documented malaria infections prior to recruitment varied from 4–58 (21.2±12.9) months for those known to have been infected with *P. falciparum* and 7–39 (20.6±10.1) months for those known to have been infected with *P. vivax*.

Of the 76 rural subjects included at enrolment, 49 (64.5%) were seen again at 3 months, 44 (57.9%) at 6 months and 51 (67.1%) at 12 months. All city individuals were re-sampled 3 months later.

None of the subjects were infected with *P. falciparum* or *P. vivax* - as determined by blood film examination and PCR - at any visit. However, one of 76 rural subjects demonstrated a significant increase in antibody titre during the study (but only to one antigen, PfSE) suggesting that this individual may have experienced a recent malaria infection, even though they did not report being ill. The three groups did not differ significantly by age or sex.

**Figure 1:**
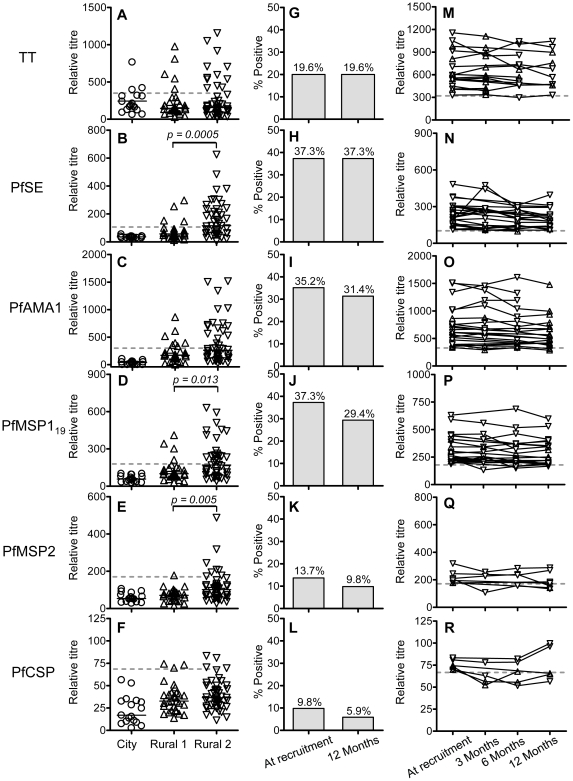
Antibody responses to *P. falciparum* antigens and tetanus toxoid. Antibody titres against tetanus toxoid (A) and *P. falciparum* antigens (B–F) among City naïve (*circle*), Rural 1 (*triangle*) or Rural 2 (*inverted triangle*) subjects at the time of recruitment were determined by indirect ELISA. Each symbol represents the antibody titre of one individual. Solid lines show the median antibody titres in each group. The Mann Whitney U test was used to analyse differences in the levels of antibodies or memory B cells among groups. Figures G–L show the percentages of all rural (i.e. Rural 1 plus Rural 2) subjects who had antibody titres above the cut-off for each antigen at the time of recruitment and 12 months later. Fischer's exact test was used to analyse differences in the proportion of seropositives at recruitment compared to 12 months later but no significant differences were observed. The antibody titres for each seropositive subject over the 12 months of the study are shown in figures M–R. Dotted lines show cut-off values calculated from a mixture model as described in materials and methods.

Antibody levels against tetanus toxoid (TT) were measured by indirect ELISA. There was no significant difference among the groups in the overall levels of antibodies to TT (Fig. 1A) but only 23.7% of the rural subjects (Rural 1 + Rural 2, n = 76) were seropositive for TT at the time of recruitment. Among the rural individuals (Rural 1 + Rural 2) who had blood collected at recruitment and 12 months later (n = 51), the seropositivity rate for TT (19.6%) did not differ between the two time points (Fig. 1G). The individual data for the rural subjects who were seropositive for TT at the time of recruitment and had blood collected more than one time point (n = 17) are shown in Figure 1M.

### Antibody responses to PfSE

Relative antibody titres to PfSE were measured, at the time of enrolment and at subsequent follow-up. Among the Rural 1 population, 4 (13.3%) individuals had antibody responses above the cut-off, indicating that they had, in fact, been exposed to malaria (Fig. 1B). Among Rural 2 subjects, 25 (54.4%) individuals were seropositive for PfSE. The proportion of seropositives in the Rural 2 group was significantly higher than in the Rural 1 group (p = 0.0003; Fisher's exact test).

There was no difference in the levels of anti-PfSE antibodies between subjects who had been infected with *P. falciparum* only or infected with *P. vivax* only (Fig. S1). Overall, the Rural 2 group had levels of anti-PfSE antibodies that were significantly higher than the Rural 1 group (p = 0.0005; Mann Whitney U test). There was no correlation between the levels of anti-PfSE antibodies and age of the subjects or the number of previous malaria episodes they had experienced (data not shown).

Among the rural subjects (Rural 1 + Rural 2) who had blood collected at the beginning and at the end of the study (n = 51), 19 (37.3%) were seropositive for PfSE at the time of recruitment and all of these remained positive 12 months later (Fig. 1H). The individual data for the rural subjects who were seropositive at the time of recruitment and had blood collected at more than one time point (n = 25) are shown in Figure 1N.

### Antibody responses to defined *P. falciparum* antigens

Antibody responses to recombinant malaria antigens PfAMA-1, PfMSP-1_19_, PfMSP-2 and PfCSP were examined by indirect ELISA. PfAMA-1, PfMSP-1_19_ and PfMSP-2 are antigens of blood stage merozoites. PfCSP is the major surface protein on the surface of sporozoites, the infective stage of the malaria parasite. All of these antigens are key *P. falciparum* vaccine candidates. City naive subjects were seronegative to all *P. falciparum* antigens tested (Fig. 1C–1F). Among the thirty rural individuals with no known episodes of malaria infection in the past 6 years (Rural 1), 7 (23%), 4 (13%), 1 (3%) and 3 (10%) subjects had positive antibody titres against PfAMA-1, PfMSP-1_19_, PfMSP-2 and PfCSP, respectively (Fig. 1C–1F). Seropositivity to individual malaria antigens, and to PfSE, was not significantly correlated (data not shown) and, overall, 10 (33%) Rural 1 subjects were seropositive to one or more *P. falciparum* antigens.

The frequencies of antibody responses to PfAMA-1, PfMSP-1_19_, PfMSP-2 and PfCSP in the previously infected (Rural 2) group were 17 (37%), 22 (48%), 7 (15%) and 4 (9%), respectively. Again, seropositivity to individual malaria antigens was not significantly correlated (data not shown) and, overall, 30 (65%) Rural 2 subjects were seropositive to one or more *P. falciparum* antigens (schizont extract and/or recombinant proteins). The proportion of individuals in the Rural 2 and Rural 1 groups who were seropositive for recombinant malaria antigens was not significantly different, except that a higher proportion of Rural 2 were seropositive for PfMSP-1_19_ (p = 0.003; Fisher's exact test).

The titres of antibodies to PfMSP-1_19_ and PfMSP-2 were significantly higher among Rural 2 subjects than among Rural 1 subjects but among the Rural 2 group the levels of antibodies to individual *P. falciparum* antigens were not different between previously *P. falciparum*- and *P. vivax*- infected subjects (data not shown). Moreover, the titres of antibodies against *P. falciparum* antigens in some Rural 2 subjects were at least as high as those of the positive control of pooled adult African sera. There was no detectable antibody response to the carrier proteins used in the production of recombinant antigens (data not shown).

Several subjects reporting prior infection only with *P. vivax* had antibodies to *P. falciparum* antigens. Of the 14 individuals with recorded *P. vivax* infections but no recorded *P. falciparum* infections, 8 (57%) had antibodies to at least one *P. falciparum* antigen. This is consistent with results of previous studies [17],[18],[19] and may reflect an undiagnosed prior infection with *P. falciparum* or cross-reactivity of antibodies to the two parasite species [17],[20],[21],[22].

As shown in Fig. 1I–1L, most of the subjects who were seropositive at recruitment and who were tested again 12 months later remained seropositive. The individual data for the rural subjects who were seropositive for the different malaria antigens at the time of recruitment and had blood collected at more than one time point are shown in Fig. 1O–1R. At an individual level, titres of antibodies against PfAMA-1, PfMSP-1_19_ and PfMSP-2 were significantly correlated with titres of anti-PfSE Abs (data not shown). No correlations between age and the antibody titres against individual malaria antigens were found (data not shown).

### Antibody responses to *P. vivax* antigens

**Figure 2:**
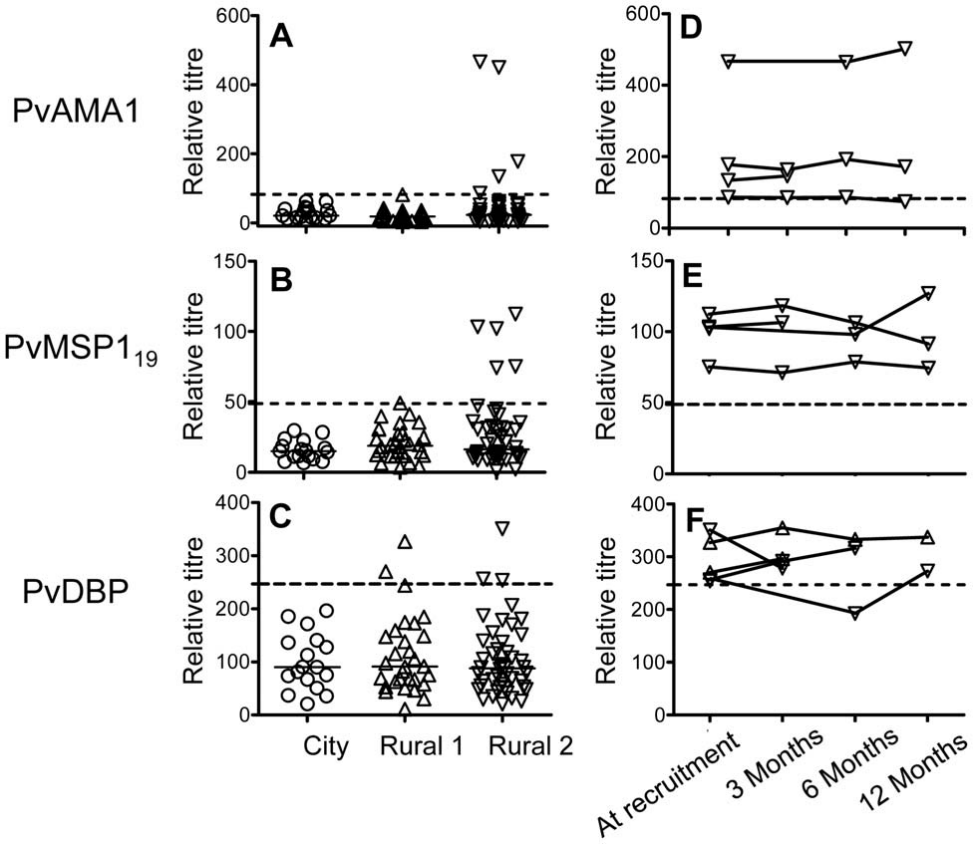
Antibody responses to *P. vivax* antigens. A–C show antibody titres against *P. vivax* antigens among City naïve (*circle*), Rural 1 (*triangle*) or Rural 2 (*inverted triangle*) subjects at the time of recruitment. Each symbol represents the antibody titre of one individual. Solid lines show the median antibody titres in each group. The titres of antibodies of seropositive rural (i.e. Rural 1 plus Rural 2) subjects at the time of recruitment and at each time point during the 12 months of study are shown in figures D–F. Dotted lines show cut-off values calculated from a mixture model as described in materials and methods.

We also investigated the antibody responses to *P. vivax* antigens, PvAMA-1, PvMSP1_19_ and PvDBP by ELISA, all of which are *P. vivax* blood stage antigens and are key vaccine candidates. City naïve subjects were seronegative to all *P. vivax* antigens (Fig. 2A–2C). Among Rural 1 subjects, none were seropositive to PvAMA-1 (Fig. 2A), one (3%) had a borderline positive titre to PvMSP-1_19_ (Fig. 2B) and two (6.7%) were seropositive to PvDBP (Fig. 2C). Of the Rural 2 subjects, 5 (11%), 5 (11%) and 3 (6.5%) were seropositive to PvAMA-1, PvMSP-1_19_ and PvDBP, respectively. Overall, 8 (17.4%) Rural 2 subjects were seropositive to one or more *P. vivax* recombinant antigens. Of the 20 subjects known to have been previously infected with *P. vivax* (*P. vivax* only or both *P. vivax* and *P. falciparum*), 5 (25%) were seropositive to one or more *P. vivax* antigens and 15 (75%) were seronegative. The proportion of subjects who were seropositive to *P. vivax* antigens did not differ significantly between the Rural 1 and Rural 2 groups. Similarly, no significant differences in anti-*P. vivax* antibody titres were observed between the Rural 1 and Rural 2 groups, although the power of this analysis was poor due to the very low numbers of seropositive subjects. Antibody responses to *P. vivax* antigens were not different between subjects known to have been infected with *P. falciparum*- and those known to have been infected with *P. vivax* (data not shown). Most of the subjects who were seropositive to *P. vivax* antigens at the time of recruitment and who had samples collected at more than one time point remained seropositive over the course of the study and there was no evidence of declining titres (Fig. 2D–2F).

### Avidity of antibodies to PfAMA-1 and PfMSP-1_19_

**Figure 3:**
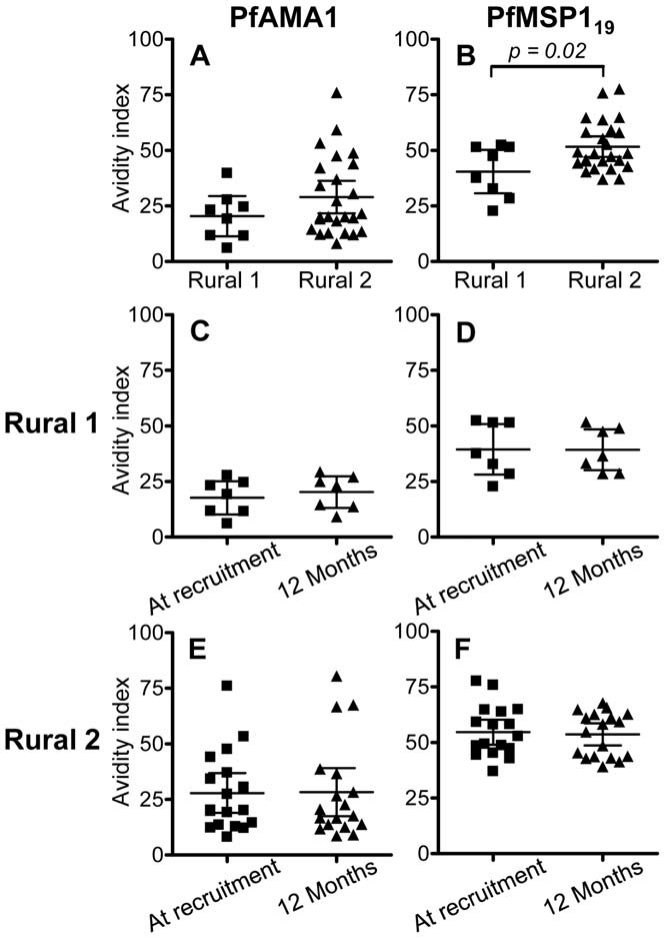
Avidity indices for anti-PfAMA-1 and PfMSP-1_19_ antibodies. Avidity indices for PfAMA-1 (A) and PfMSP-1_19_ (B) at the time of recruitment were compared between Rural 1 and Rural 2 groups. Avidity indices for antibodies to both antigens were compared at the time of recruitment and at 12 months later for Rural 1 (C and D) and Rural 2 (E and F) subjects. Lines show the mean (95% CI) for each group. An unpaired Student's *t*-test test was used to analyse differences between rural groups. A paired *t*-test was used to confirm that there are no differences between indices at time of recruitment and 12 months later.

Antibody avidity tends to increase over time as a result of somatic mutation in the immunoglobulin-encoding genes of germinal centre B cells and in response to increasing competition between B cell clones for diminishing amounts of antigen [23],[24]. To determine whether the avidity of the anti-malarial antibody response changed over time, or whether antibody avidity was associated with durability of antibody responses, the avidity indices of anti-PfAMA-1 and anti-PfMSP-1_19_ antibodies were determined for all those individuals (as defined in Figure 1) who were seropositive to one or other antigen at the time of recruitment. Overall, avidity indices for antibodies to both antigens were higher in Rural 2 group than in Rural 1 group (Fig. 3A and 3B), and this difference was statistically significant for antibodies to PfMSP-1_19_. However, there was no detectable change in the avidity of antibodies to either antigen in either group over the 12 months of the study (Fig. 3C–3F).

### Longevity of antimalarial antibodies

**Figure 4:**
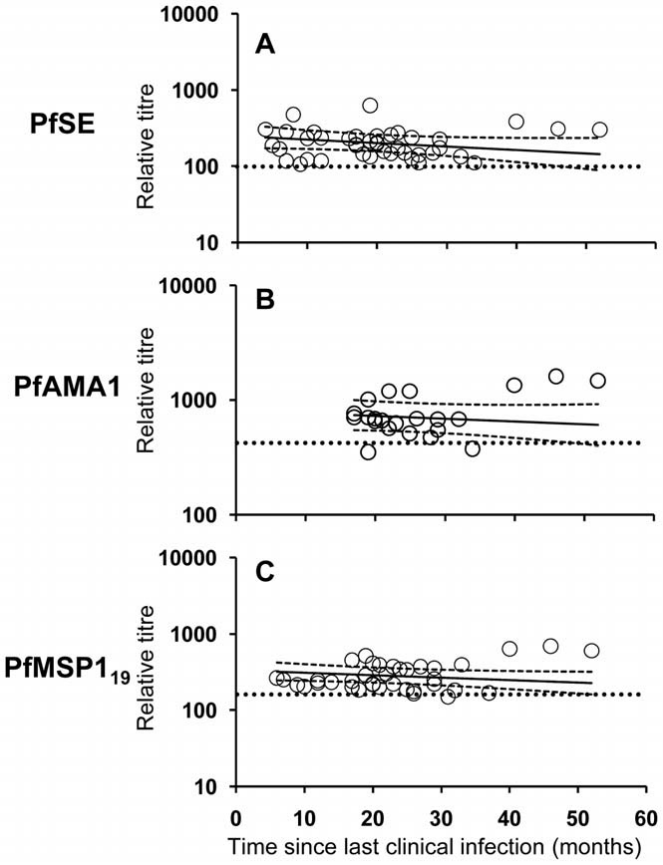
Longevity of anti-malarial antibody responses. The titres of antibodies specific to PfSE (A), PfAMA-1 (B) and PfMSP-1_19_ (C) in relation to time since last clinical infection in *P. falciparum* exposed individuals (Rural 2 only) were determined by analyzing longitudinal data with a mixed-effects model. Each symbol represents the antibody level at each time point of one individual. The regression analysis was adjusted for inclusion of multiple data points from the same individual. Solid lines represent best fit regression lines estimating the rates of decline of antibody concentrations over time and the dashed lines represent the 95% CI. Horizontal dotted lines indicate the cut-off as defined in Materials and Methods and Figure 1.

To determine the longevity of the antimalarial antibody responses, we analysed the change in concentrations of antibodies to PfSE, PfAMA-1 and PfMSP-1_19_ in relation to time (in months) since the last documented malaria episode (Figure 4). The half-life of the antibody response was analysed separately for each antigen using data from Rural 2 subjects who were seropositive at the time of enrolment and for whom follow-up samples were obtained (PfSE n = 14; PfAMA1 n = 8; and PfMSP-1_19_ n = 12) in a repeated measurements analysis including multiple data points from the same subjects. Subjects known to be infected with *P. vivax* but not known to have been infected with *P. falciparum* were not included in this analysis in order to ensure specificity to *P. falciparum*.

Mixed-effects regression models revealed very low rates of decline (converted to years) in anti- PfSE, PfAMA-1 and PfMSP-1_19_ antibody concentrations over time and statistically, these rates could not be distinguished from zero (Table 2). The best estimates of half-lives were: 5.5 years for PfSE, 10.4 years for PfAMA-1 and 7.6 years for PfMSP-1_19_, respectively but, in each case, the 95% CI included infinity. Pooled regression analysis of data for antibodies to PfAMA-1 and PfMSP-1_19_ also yielded a rate of decline that was not statistically significant from zero. Inclusion of anti-PfSE antibody data in the pooled regression analysis resulted in a marginally significant rate of decline equivalent to a half-life of 6.4 years (95% CI = 3.22, 650.48; *p = 0.048*). These analyses suggest that antibody responses to malaria are stably maintained in this population.

**Table 2:** Longevity of antibody and memory B cell responses to *P. falciparum* antigens*.

ANTIBODY					
Antigen	No. of subjects	No. of observations	Annual decline in log titre (95% CI)	Antibody half-life (95% CI)	p-value
PfSE	14	41	−0.1264 (−0.2809, 0.0282)	5.49 (2.47, ∞)	0.109
PfAMA1	8	23	−0.0669 (−0.2009, 0.0671)	10.36 (3.45, ∞)	0.328
PfMSP1_19_	12	37	−0.0916 (−0.1959, 0.0126)	7.56 (3.54, ∞)	0.085
PfMSP1 + PfAMA1	14	60	−0.0426 (−0.1672, 0.082)	16.27 (4.15, ∞)	0.503
All	17	101	−0.1082 (−0.2153, −0.0011)	6.41 (3.22, 650.48)	***0.048***

B CELL MEMORY					
Antigen	No. of subjects	No. of observations	Annual decline in log titre (95% CI)	Antibody half-life (95% CI)	p-value
PfAMA1	13	26	0.0194 (−0.3858, 0.4245)	∞ (1.8, ∞)	0.925
PfMSP1_19_	10	19	−0.0689 (−0.5872, 0.4494)	10.06 (1.18, ∞)	0.794
Both	15	45	−0.0928 (−0.3959, 0.2102)	7.47 (1.75, ∞)	0.548

*: Results from a multilevel model allowing for random patient effects.

### B cell memory responses to *P. falciparum* and *P. vivax* antigens

**Figure 5:**
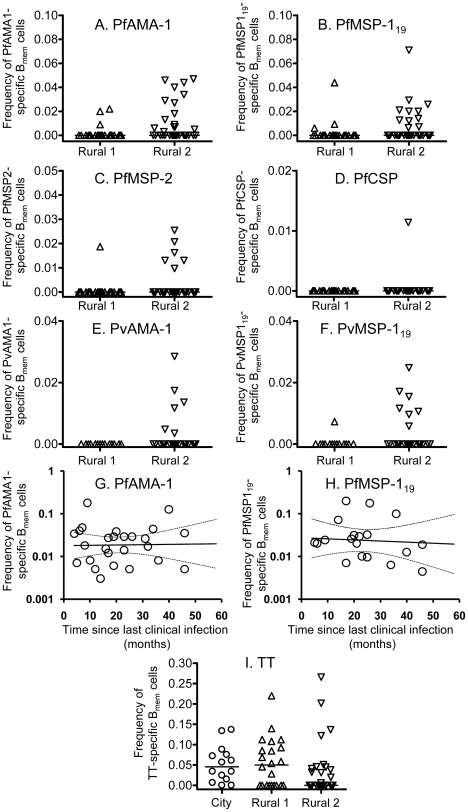
B cell memory responses to malaria antigens and tetanus toxoid. B cell memory responses to *P. falciparum* antigens (A–D), *P. vivax* antigens (E and F) and tetanus toxoid (I) at the time of recruitment were determined by ELISPOT assay and are presented as the percentage of all IgG-secreting cells that are specific for each malaria antigen. Each symbol represents the memory B cell numbers for one individual. The longevity of the memory B cell responses specific to PfAMA-1 (G) and PfMSP-1_19_ (H) were determined by analyzing longitudinal data with a mixed-effects model. Solid lines represent best fit regression lines estimating the rates of decline of memory B cell numbers over time and the dashed lines represent the 95% CI.

We next enumerated memory B cells to malaria antigens and to TT using a highly sensitive ELISPOT protocol [25]. The number of subjects available for analysis was limited by availability of cryopreserved PBMCs. Antigen-specific memory B cell frequencies are presented as a percentage of the total number of IgG-secreting cells. Frequencies of TT-specific memory B cells were similar among the three study groups (Fig. 5I). No spots were detected for any individual when cells were tested against the irrelevant control protein (keyhole limpet hemocyanin) and no malaria-specific spots were observed in samples from the City naïve group (data not shown).

At the time of recruitment, of the 21 Rural 1 subjects whose PBMCs were available, 3 (14.2%), 3 (14.2%), and 1 (4.8%) subjects had memory B cells specific to PfAMA-1 (Fig. 5A), PfMSP-1_19_ (Fig. 5B), and PfMSP-2 (Fig. 5C), respectively. No memory B cells specific to PfCSP were found in the Rural 1 group (Fig. 5D). A much higher proportion of Rural 2 individuals had detectable memory B cells: of the 33 tested, 16 (48%), 11 (33%), 6 (18%) and 1 (3%) gave spots to PfAMA-1, PfMSP-1_19_, PfMSP-2 and PfCSP, respectively. Overall, 19 (58%) Rural 2 subjects had memory B cells to one or more *P. falciparum* recombinant antigens.

None of the 14 Rural 1 subjects tested had detectable memory B cells against PvAMA-1, and only one individual (7%) had detectable memory B cells to PvMSP-1_19_ (Fig. 5E and 5F). However, among the 26 Rural 2 individuals tested, 6 (23%) and 7 (27%) had memory B cells specific to PvAMA-1 and PvMSP-1_19_, respectively. Nine Rural 2 subjects (35%) had memory B cells specific to one or more *P. vivax* antigens.

### Stability of PfAMA-1- and PfMSP-1_19_-specific memory B cells

For the Rural 2 individuals, we then characterised the frequency of PfAMA-1- and PfMSP-1_19_-specific memory B cells in relation to time since their last documented malaria infection, using mixed-effects regression analysis (allowing for repeated measurements from individual subjects) as described above. We found that PfAMA-1- and PfMSP-1_19_ specific memory B cells were stably maintained over time (Fig. 5G and 5H). The best estimate of the rate of change in AMA-1-specific memory B cell numbers indicated no decline during follow-up, whereas the best estimate for the half-life of MSP-1_19_-specific memory B cells was 10 years (Table 2). Single and pooled regression analysis of data resulted in rates of decline that, statistically, could not be distinguished from zero. Similar observations were made for memory B cells to TT (data not shown). These results indicate that memory B cell responses to malaria antigens are stably maintained in this very low transmission area.

### Correlation between antibody titres and memory B cell frequencies

**Figure 6:**
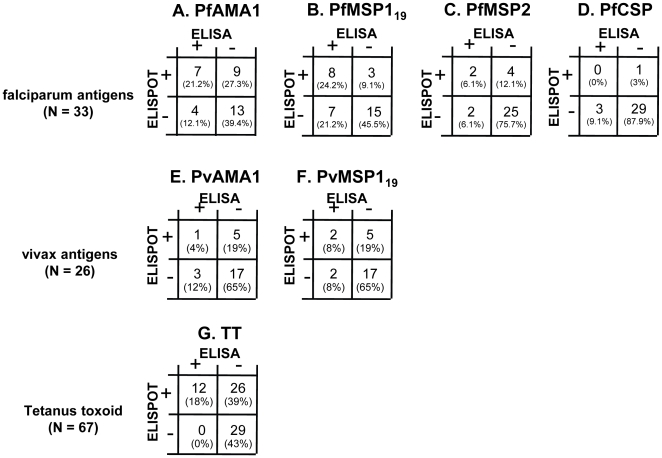
Correlation between ELISA and ELISPOT responses for each antigen. For malaria antigens, data are shown for Rural 2 subjects: 33 tested against *P. falciparum* antigens (A–D) and 26 tested against *P. vivax* antigens (E and F). For TT (G), data are shown for all subjects (City naive, Rural 1 and Rural 2) whose PBMC were available (n = 67). The number (and percentages) of subjects who were double positive (top left), ELISA positive but ELISPOT negative (bottom left), ELISA negative but ELISPOT positive (top right), or double negative (bottom right) are shown.

It was immediately evident from the TT data that circulating memory B cells could be detected in many (∼47%) seronegative individuals (Figure 6G). We therefore carried out a systematic analysis of the association between circulating memory B cells and plasma antibody titres at the individual level.

No correlation was observed between specific antibody titres and frequencies of memory B cells (data not shown). Among the 33 Rural 2 subjects for whom we had both antibody data and memory B cell responses to *P. falciparum* antigens at the time of recruitment, 7 (21%), 8 (24%) 2 (6%) and 0 (0%) had both circulating antibody and memory B cells to PfAMA-1, PfMSP-1_19_, PfMSP-2 and PfCSP respectively (Fig. 6A–6D). Four (12%), 7 (21%), 2 (6%) and 3 (9%) individuals were seropositive to the respective antigens but no B cell spots were observed whereas 9 (27%), 3 (9%), 4 (12%), and 1 (3%) were seronegative but had memory B cells to PfAMA-1, PfMSP-1_19_, PfMSP-2 and PfCSP respectively.

Of the 26 Rural 2 subjects for whom we had data on both antibody and memory B cell responses against *P. vivax* antigens at the time of recruitment, 1 (4%) and 2 (8%) individuals had both antibody and memory B cells against PvAMA-1 and PvMSP-1_19_ respectively (Fig. 6E and 6F), 3 (12%) and 2 (8%) gave positive results in ELISA but not in ELISPOT, and 5 (19%) and 5 (19%) had memory B cells but not antibodies to PvAMA-1 and PvMSP-1_19_, respectively. These results suggest that serum antibody levels alone or memory B cell frequencies alone may not fully represent the humoral immune response to malaria parasites.

Different individuals had different patterns of antibody and memory B cell responses to the various malaria antigens. Table 3 shows the heterogeneity of such responses in all Rural 2 subjects at recruitment. In a number of cases where antigen-specific antibodies were detected, the frequencies of memory B cells were below the limit of detection (Subjects 16, 17 and 29). Likewise, in several subjects where antigen-specific antibodies were not detected, stable frequencies of antigen-specific memory B cells were observed (e.g. Subjects 27, 31 and 33).

**Table 3:** Patterns of antibody and memory B cell responses to malaria antigens and tetanus toxoid in 46 Rural 2 subjects at recruitment.

Subject	PfSE[a]	*P.falciparum* antigens								*P.vivax* antigens				Tetanus toxoid	
		PfAMA1		PfMSP1_19_		PfMSP2		PfCSP		PvAMA1		PvMSP1_19_			
	ELISA	ELISA[b]	ELISPOT[c]	ELISA	ELISPOT	ELISA	ELISPOT	ELISA	ELISPOT	ELISA	ELISPOT	ELISA	ELISPOT	ELISA	ELISPOT
**Tested against both ** ***P.falciparum*** ** and ** ***P. vivax antigens***															
**1**	x	x		x	x	x									x
**2**	x		x	x						x		x	x	x	x
**3**				x	x								x		
**4**	x														
**5**															x
**6**	x										x		x	x	x
**7**															x
**8**															x
**9**															
**10**		x	x	x											
**11**	x	x		x											
**12**	x												x		
**13**	x	x	x	x							x				
**14**	x	x		x	x					x					
**15**			x											x	
**16**	x			x		x									
**17**		x													
**18**										x	x	x	x		x
**19**															x
**20**	x	x	x	x	x				x		x		x		x
**21**														x	x
**22**	x	x	x	x		x	x	x		x		x			
**ELISPOT tested against ** ***P. falciparum*** ** antigens only**															
**23**	x		x	x	x	x	x	x			N/D		N/D	x	
**24**				x	x						N/D		N/D	x	
**25**											N/D		N/D		
**26**	x	x									N/D		N/D	x	
**27**	x	x			x			x			N/D		N/D	x	x
**28**			x		x		x				N/D		N/D	x	
**29**	x			x							N/D		N/D		
**30**	x	x		x							N/D		N/D		
**31**	x	x		x	x						N/D		N/D	x	
**32**			x		x		x				N/D		N/D		
**33**	x	x	x	x	x		x				N/D		N/D		
**34**			x				x				N/D		N/D	x	x
**35**											N/D		N/D		
**ELISPOT tested against ** ***P. vivax*** ** antigens only**															
**36**	x		N/D		N/D	x	N/D		N/D						
**37**			N/D		N/D		N/D		N/D		x		x		
**38**	x		N/D	x	N/D		N/D		N/D						x
**39**	x	x	N/D	x	N/D		N/D		N/D		x	x		x	
**Tested for antibodies only**															
**40**			N/D		N/D		N/D		N/D		N/D		N/D		N/D
**41**	x	x	N/D	x	N/D	x	N/D	x	N/D	x	N/D	x	N/D	x	N/D
**42**			N/D	x	N/D		N/D		N/D		N/D		N/D		N/D
**43**	x	x	N/D	x	N/D		N/D		N/D		N/D		N/D		N/D
**44**			N/D		N/D		N/D		N/D		N/D		N/D		N/D
**45**	x	x	N/D	x	N/D	x	N/D		N/D		N/D		N/D		N/D
**46**	x		N/D		N/D		N/D		N/D		N/D		N/D		N/D

[a] x indicates positive response to each antigen.

[b] Cut-off values were determined from a mixture model.

[c] A positive response was defined if the average number of spots from triplicate wells being greater than at least two times the average number of spots from the media negative control.

N/D = Not done.

## Discussion

Antibodies are critical in protection against blood stage malaria infection through numerous, diverse mechanisms [2],[3]. In murine malaria infections, B cells are required not only for the production of protective Abs, but also for the development of T cell helper function [26]. However, the development and persistence of B cell memory responses following malaria infection has repeatedly been called into question [27]. It is widely perceived that antibody titres rapidly decline in the absence of re-infection or when individuals leave an endemic area. It is notable that most of the studies reporting short-lived antibody responses have been conducted at or following the time of acute infection, and these infections were terminated by effective antimalarial drug therapy and that these were often observations in children [13]–[16],[28]. It is not clear, from these studies, whether the rapid decline of antibody concentrations observed in children is related to removal of antigen by chemotherapy, by consumption of antibodies and formation of antigen-antibody complexes during parasite clearance or due to limitations in the ability of the bone marrow compartment to support differentiation and/or survival of plasma cells [29],[30]. However, the results of a recently published study in healthy, Gambian children in which antibody titres were found to decline more slowly both in older children and in children with persistent asymptomatic malaria infection suggests that both antigen persistence and immunologic maturity may be important in determining the longevity of the serum antibody responses [15].

One explanation for these observations might be that short-lived antibody responses are the result of induction of short-lived, but not long-lived, plasma cells following acute malaria infection. In murine models of malaria infection, primary *P. chabaudi* infection leads to expansion of short-lived, immature B220^+^ splenic plasma cells however secondary infection is accompanied by apparently normal emergence of a larger population of fully mature (Ig^hi^, CD138^hi^, CD9^+^, B220^−^), terminally-differentiated B220- plasma cells in the bone marrow [31], indicating that memory B cells are efficiently induced by primary infection and are fully able to differentiate into long-lived plasma cells on secondary exposure to antigen. Similar studies have not been reported, to date, in humans but we were able to take advantage of a very particular epidemiological situation in rural Northern Thailand to examine the natural history of the anti-malarial B cell memory response.

In our study area, both *P. falciparum* and *P. vivax* are endemic but transmission is kept at extremely low levels by an assiduous malaria surveillance and control programme in which all detected infections are recorded and effectively treated [32]. We have thus been able to recruit a cohort of individuals whose malaria infection history over the previous 6 years are known in considerable detail and have been able to follow these individuals for a period of 12 months to observe both long- and short-term changes in their adaptive immune response to malaria. Furthermore, we were able to recruit a cohort of individuals from the same community with no evidence of malaria infection in the past 6 years , and a cohort of known malaria naives from the city of Chiang Mai, where malaria transmission was eliminated more than 30 years ago (Suwonkerd W; The Ministry of Public Health; personal communication). The very low levels of malaria transmission reported in the Muang Na area [33] are confirmed by our finding that none of the study subjects were found to be infected with malaria parasites (detectable by blood film or PCR) at any point during the study, none of them showed any clinical signs of malaria infection and only one individual showed boosting of antibody responses (and against only 1 malaria antigen) during the study. In addition, anti-malarial antibody responses were not correlated with age, indicating that there is likely to be little or no effective acquired immunity to malaria in this population.

Nevertheless, some of the rural village residents with no record or recollection of malaria infection in the past 6 years (Rural 1) appeared to have experienced malaria infections at some time in their lives as shown by seropositivity to malaria antigens in ELISA and positive B cell ELISPOTs. Given the lack of evidence for acquired protective immunity in this population, and since we found no evidence of asymptomatic malaria infections, it is unlikely that these individuals had experienced undiagnosed malaria infections and thus the presence of antibodies and B cell memory responses in this group suggests that anti-malarial seropositivity can be maintained for many years in the absence of reinfection.

Overall, the prevalence and magnitude of antimalarial antibody and memory B cell responses compared favourably with the anti-tetanus responses. Although the frequencies of tetanus-specific memory B cells tended to be somewhat higher than the frequencies of malaria-specific memory B cells, the prevalence of antibodies to the *P. falciparum* schizont extract, PfMSP-1_19_ and PfAMA-1 was in fact higher than for tetanus. Thus, despite the fact that the anti-tetanus response is likely induced by a very potent vaccine and boosted by environmental exposure or revaccination (which is routinely given during pregnancy), humoral immune responses to tetanus do not appear to be particularly more robust than those induced by infrequent natural exposure to malaria. Moreover, frequencies of malaria-specific memory B cells in Thai adults were similar to frequencies of diphtheria-specific memory B cells in UK adults (J. Palomero-Gorrindo and J. Hafalla; unpublished data). Therefore, frequencies of malaria-specific memory B cells seem to be of the same order of magnitude as responses to commonly used vaccine antigens [34],[35]. Of note, Buisman et al [35] also found rather higher frequencies of memory B cells to tetanus toxoid than to other antigens.

As the time since last detected malaria infection was known for the Rural 2 group we were able to obtain estimates of the rate of decline of both serum antibodies and circulating memory B cells. Importantly, and in marked contrast to previously published data from African children [14], there was no evidence of any significant decline in either antibody titres or memory B cell responses to PfSE, PfAMA-1 or PfMSP-1_19_ over periods of more than 5 years since the last known malaria infection. One reason for this discrepancy may be that previous studies have characterised the decay of the antibody response in the first few days or weeks after resolution of an acute malaria infection [14],[15] which is likely to capture the initial very rapid decay in antibody titre associated with contraction of the pool of short-lived plasma cells whereas in this study, where the most recent malaria infection occurred many months or years ago, we are capturing the long term “maintenance” phase of the antibody response [36].

Our best estimates of the half-life of this maintenance phase of antibody responses to malaria antigens ranged from ∼5 to ∼16 years, putting them in the same range as the half-lives recently estimated for nonreplicating antigens such as tetanus or diptheria toxoids [37]. However, the 95% confidence intervals for the half-lives of these responses all included infinity, suggesting that antibody responses to malaria may in fact be much more stable than those to nonreplicating antigens and may be maintained in a manner that is more similar to that of antiviral responses [37]. Similar estimates were obtained for the half lives of malaria-specific memory B cell responses – indicating that the circulating memory B cell pool is extremely stable - which is consistent with data from the *P. chabaudi* mouse model that malaria infection induces long-lived antibody responses as well as memory B cells [31]. However, whilst these analyses are consistent with a long half life for antimalarial antibody and memory cell responses, given the very wide confidence intervals around the our estimates, data from a larger cohort of study subjects is required to obtain definitive half lives for these responses.

However, it is important to note that 11 subjects (24%) who were known to have been infected with malaria had no detectable circulating memory B cells and/or antibodies and less than 50% of subjects tested had either antibodies or circulating memory B cells to PfMSP-2, PfCSP, PvAMA-1 or PvMSP-1_19_. Given that everyone in the study had experienced their most recent malaria infection at least 4 months before recruitment and that the genotypes of their infecting parasites are unknown, we cannot tell whether these seronegative individuals had completely failed to make a humoral response to malaria, whether they had made antibodies to polymorphic epitopes that did not cross-react with the antigens used in our assays or whether they had developed only very short-lived responses. In a previous study, in a higher malaria transmission area, very short-lived antibody responses to malaria were particularly associated with younger individuals who (presumably) had had the fewest number of malaria infections [15] suggesting that the long-lived responses seen in many of our study subjects may develop only after they have experienced a number of malaria infections. Nevertheless, given the very low levels of malaria transmission in our study area, the number of infections required to develop long-live antibody responses is likely to be quite small.

Since the pharmacological half-life of a human IgG molecule is around 21 days [38], long term maintenance of IgG titres indicates either ongoing secretion of antibodies from plasma cells or memory B cell differentiation in response to inflammatory stimuli; there is still no clear consensus on whether persisting specific antigen is required for this process [39] or not [40],[41]. During inflammation, IFN-γ induces plasmablasts to express the chemokine receptor CXCR3, promoting their migration into inflamed tissues [42] and thereby maximising antibody production at sites of infection. Resolution of inflammation leads to loss of survival signals, and these short-lived plasma cells die *in situ*. However, in the absence of prolonged antigenic stimulation, plasmablasts express another chemokine receptor CXCR4 allowing them to migrate to the bone marrow [43]. It is possible, therefore, that short term fluctuations in serum antibody concentrations may occur in response to infection with memory B cells being stimulated to differentiate into short-lived plasma cells, secrete immunoglobulins and then die. However, in the circumstances of lack of frequent re-exposure to malaria infection (such as in this study), a larger proportion of plasmablasts may differentiate into long-lived plasma cells which maintain the level of antibodies over time. Such a scenario does, of course, beg the question as what would happen to long-lived plasma cells and memory B cells under conditions of repeated or persistent malaria infection.

Immediately after malaria immunisation in humans the frequency of antigen-specific memory B cells is positively correlated with antibody titres [44], suggesting (not surprisingly) that induction of antibody secreting cells and memory B cells is linked. However, the lack of correlation that we observed between antibody titres and memory B cell responses months or years after exposure to malaria antigens confirms recent findings for anti-viral responses [37] and is consistent with accumulating experimental data from animals [45],[46] and data from therapeutic B cell depletion in humans [47],[48] showing that depletion of circulating memory B cells does not affect antibody titres, at least in the short-term. Collectively, these data indicate that although both long-lived plasma cells and memory B cells can be stably maintained the two populations are independently regulated and that activation of circulating memory B cells may not be required for maintenance of serum antibody titres.

High affinity antibodies are expected to play an important role in the humoral immune response. The avidity indices of antibodies against PfAMA-1 and PfMSP-1_19_ did not change during the 12 months of study; this is not surprising since there was no evidence of reinfection of any of the subjects during the follow-up period which might have driven further avidity maturation. However the avidity of anti-PfMSP-1_19_ antibodies was significantly higher among Rural 2 subjects than among Rural 1 individuals, supporting the notion that the Rural 2 population had had more frequent exposure to malaria parasites than the Rural 1 group.

In summary, we conclude that B cell memory responses to malaria are effectively induced and maintained – in a significant proportion of individuals - in areas of low malaria transmission. (This is, of course, an entirely separate issue from whether these particular antibodies confer protective immunity to malaria; whilst there is strong evidence to suggest that malaria-immune individuals have very effective antimalarial antibody responses [49] the antigenic targets of protective antibodies are still very poorly defined. Whilst it is possible that the subjects in this study with long-lived humoral responses to malaria antigens might be protected from reinfection, this issue was not directly addressed in this study). Although it remains possible that persistent and repeated malaria infections in areas of very high endemicity may eventually lead to B cell anergy or clonal exhaustion [50], the fact that individuals in these areas develop high titres of antimalarial antibodies and become resistant to high density malaria infections and clinical symptoms argues against this as a major impediment to the development of effective immune responses. Finally, our results are highly encouraging for vaccine developers since they imply that – once induced – anti-malarial immune responses are likely to be long-lived even in the absence of frequent boosting.

## Materials and Methods

### Study area and subjects

Study subjects were either long-term adult residents of Muang Na, a village in a low malaria transmission area in the Chiang Dao region of northern Thailand, near the border with Myanmar, or were permanent adult residents of the city of Chiang Mai where malaria transmission does not occur. Ethical approval for the study was obtained from the Research Institute for Health Sciences, Chiang Mai University, from the Ministry of Public Health, Thailand and from the London School of Hygiene and Tropical Medicine, UK. Written informed consent was obtained prior to enrolment in the study.

Subjects were interviewed to ascertain their previous malaria exposure. Residents of Chiang Mai were selected on the basis that they had not travelled to, or lived in, malaria endemic areas. In Muang Na, dates and species (*P. falciparum*, *P. vivax* or both) of malaria infections were confirmed from the records of the Office of Vector Borne Disease Control in the Department of Communicable Diseases Control at the Ministry of Public Health, which maintains detailed records of all malaria cases detected by active or passive case detection and during periodic population surveys as described in detail elsewhere [51].

Venous blood was collected in acid citrate dextrose on the day of recruitment, and again 3 months later for City naïve subjects and 3, 6 and 12 months after recruitment for rural subjects. Giemsa-stained blood films were examined for the presence of malaria parasites. Blood samples from each subject were checked for subpatent malaria parasitaemia by PCR. DNA was isolated using FlexiGene DNA extraction kits (Qiagen®) according to the manufacturer's protocol and subjected to nested PCR for *P. falciparum* and *P. vivax* as described previously [52].

As HIV infection may have an effect on immunological parameters, all subjects were tested for HIV infection (presence of anti-HIV antibodies by gel particle agglutination assay) at the time of recruitment and at the end of the study (3 months after recruitment for city subjects and 12 months after recruitment for other groups); subjects received pre- and post-test counselling from trained HIV counsellors and HIV-infected individuals were given access to the National Antiretroviral Programme. Data from HIV-infected subjects were excluded from the analysis.

### Antigens

*P. falciparum* circumsporozoite protein (PfCSP) [(NANP)_4_] and *P. falciparum* merozoite surface protein-2 (PfMSP-2) were a gift from J.E. Tongren (Centre for Disease Control and Prevention, Atlanta, GA, USA). The 19kDa fragments of *P. falciparum* and *P. vivax* MSP-1 (PfMSP-1_19_ and PvMSP-1_19_) were gifts from A. Holder (National Institute of Medical Research, London, UK) and the proteins were expressed as described [53]. *P. falciparum* apical membrane antigen-1 (PfAMA-1) was a gift from R.F. Anders (LaTrobe University, Victoria, Australia); the equivalent *P.vivax* antigen (PvAMA-1) was a gift from B. Farber and A. Thomas (Biomedical Primate Research Centre, Rijswik, Netherlands). *P. vivax* duffy binding protein (PvDBP) was a gift from L.H. Carvalho Centro de Pesquisas René Rachou, Fundação Oswaldo Cruz, Belo Horizonte, MG, Brazil). Since Thai populations are routinely vaccinated with tetanus toxoid (TT), antibody responses to TT were included as a positive control. TT was obtained from the National Institute of Biological Standards and Control (Health Protection Agency, Hertfordshire, UK). Keyhole limpet haemocyanin (KLH) was from Thermos Fisher Scientific (Northumberland, UK).

Continuous cultures of *P. falciparum* (3D7 strain were maintained in the laboratory [54] and were periodically shown to be free from Mycoplasma contamination by polymerase chain reaction (PCR) (Venor® GeM, Minerva Biolabs). Mature schizonts were obtained by gradient centrifugation over 60% Percoll (Amersham Biosciences), adjusted to a concentration 1×10^8^ schizont-infected red blood cells (iRBC)/ml and exposed to three freeze/thaw cycles to obtain *P. falciparum* schizont extract (PfSE).

### Enzyme-linked immunosorbent assay (ELISA)

Plasma antibody levels were detected by indirect ELISA, as described [55]. Briefly, Immulon 4HB (Dynatech) or Maxisorb (Nunc) plates were coated with antigen (at a concentration equivalent to 10^5^ iRBC/ml for PfSE, 0.5 µg/ml for *Plasmodium*-derived antigens and TT) in bicarbonate buffer (pH 9.6) overnight at 4°C. Plates were blocked with PBS containing 1% non-fat milk powder. Diluted plasma samples (1∶200 for PfCSP, 1∶1000 for PfSE, PfMSP-1_19_, PfMSP-2, PvMSP-1_19_ and PvDBP; 1∶2000 for PfAMA-1, PvAMA-1 and TT) were incubated in dubplicate. Plates were subsequently developed with anti-human IgG horseradish peroxidase conjugate (Caltag Laboratories, Invitrogen, Paisley, UK) followed by *o*-phenylenediamine substrate (Sigma). The enzyme reaction was terminated with sulphuric acid (2N) and absorbance was then read at 492 nm on a Spectra MR plate reader (Dynex Technology).

Antibody levels were determined by comparison to a standard curve (derived by serial dilution of a pool of hyperimmune plasma collected in The Gambia, which was given an arbitrary value of 1,000 units/ml of anti-PfSE Abs) on each plate, as described previously [12].

### Preparation of peripheral blood mononuclear cells (PBMCs)

PBMCs were separated from citrated blood by gradient centrifugation over Ficoll-Hypaque (Amersham Biosciences). Contaminating erythrocytes were removed by incubating with lysis buffer (0.15 M NH_4_Cl, 10 mM KHCO_3_, 0.1 mM Na_2_EDTA) at RT for 5 minutes. The cells were washed twice with RPMI, resuspended in 10% human AB serum/RPMI (R10 culture medium), counted, adjusted to the required concentration and cryopreserved in 10% dimethylsulfoxide (DMSO)/foetal calf serum.

### Stimulation of cryopreserved PBMC for B cell ELISPOT assay

Cryopreserved PBMCs were quick thawed in a 37°C water bath. The cells were washed twice with warm RPMI, resuspended in R10 culture medium and added at a concentration of 1×10^6^ cells/ml to a 24 well culture plate. The cells were stimulated with medium alone or with a mixture of *Phytolacca americana* pokeweed mitogen (1/100,000 dilution; a gift from M. Causland and S. Crotty, La Jolla Institute of Allergy and Immunology, CA, USA), 6 µg/ml CpG 2006 (Qiagen/Operon), and 1/10,000 dilution of Staphylococcus Aureus Cowan (SAC) (Sigma), as previously described [25]. The culture plates were incubated in 5% CO_2_ at 37°C for 5 days.

### B cell ELISPOT assay

B cell ELISPOT assays were performed as described previously [25]. Briefly, ELISPOT plates (Millipore) were coated with donkey anti-human IgG (H+L) (Jackson ImmunoResearch), or with 1 µg/ml recombinant malaria proteins overnight at 4°C. After washing once with PBS-T and three times with PBS, 200 µl of 1% bovine serum albumin in RPMI were added to each well and incubated for 2 hours at 37°C, 5% CO_2_. Cultured PBMCs were recovered from the 24 well culture plates, washed, transferred directly to antigen-coated ELISPOT plates and incubated for 6 hours at 37°C, 5% CO_2_. After 4 washes with PBS and 4 washes with PBS-T, 100 µl of Biotin-SP-conjugated donkey anti-human IgG (Jackson Immunoresearch) were added to each well and the plates were incubated overnight at 4°C. The plates were washed, 100 µl of alkaline phosphatase-streptavidin (Vector Laboratories) was added and incubated for one hour at room temperature. After three washes with PBS-T and three washes with PBS, 100 µl of 5-bromo-4-chloro-3-indolyl phosphate/ nitro blue tetrazolium - alkaline phosphatase substrate solution (Vector Laboratories) were added to each well and the reaction was allowed to proceed for 8 minutes before being stopped with distilled water. *In vitro* restimulated PBMCs incubated overnight with an irrelevant protein, KLH, as well as PBMCs cultured without stimulation and then incubated overnight with malaria antigens were used as negative controls. Since no malaria-specific spots were detected in city naïve individuals, this group was not be used to set a cut-off for positivity. Rather, a positive ELISPOT response was defined when spots were observed in 2 or more replicate wells and where the total number of spots in the antigen-coated wells was at least twice the number observed in the negative control wells.

### Avidity assay

An enzyme immunoassay for determination of antibodies against malaria antigens was carried out as described above. Following the incubation step of sera with antigens, one duplicate set of sera was treated with 4.0 M guanidine dissociating solution (Guanidine Hydrochloride, Sigma) for 10 minutes prior to washing with PBS-T. Avidity indices were calculated as the ratio of the OD of guanidine -treated wells to the OD of the untreated wells.

### Statistical analysis

To determine whether an individual was seropositive for a particular antigen (PfSE, *Pf*- or *Pv*-derived antigens, or TT), cut-offs for positive antibody titres were calculated using a mixture model, which assumes that untransformed titres for seropositive and seronegative samples each follow a normal Gaussian distribution [56],[57]. Mann Whitney U test was used to analyse differences in the levels of antibodies or memory B cells among groups (GraphPad Prism software). Fischer's exact test was used to analyse differences in the proportion of positive individuals between Rural 1 and Rural 2 groups, as well as differences in the proportion of seropositives at recruitment compared to 12 months later. Decay rates for antibody titres and memory B cell frequencies were calculated using logarithmically transformed data from subjects who were seropositive or memory B cell positive, respectively, at recruitment. The effect of time since malaria infection was analysed using a log-linear mixed-effects regression model incorporating Gaussian random intercepts. This resulted in an estimate of the decay rate of antibody titres or memory B cell frequencies, assuming a single-exponential decay model. Half-lives were calculated from the estimated decay rate and the boundaries at 95% confidence interval obtained from the mixed-effects model. Where the decay rate is a positive value, the calculated half-life is reported as infinity. All analyses were undertaken using Stata (version 10, Statacorp LP).

## Supporting Information

Figure S1 Antibody responses against PfSE in *P. falciparum* (*square*) and *P. vivax* (*diamond*) exposed subjects. Each symbol represents the antibody titre of one individual. Dotted lines show cut-off values calculated from a mixture model as described in materials and methods. Solid lines show the median antibody titres in each group.
(0.03 MB DOC)
