# Supplementary material for: Long-Lived Antibody and B Cell Memory Responses to the Human Malaria Parasites, Plasmodium falciparum and Plasmodium vivax
Source: PLoS Pathog. 2010 Feb 19;6(2):e1000770. doi: 10.1371/journal.ppat.1000770 (PMC2824751; doi:10.1371/journal.ppat.1000770)
Supplement: Figure S1 — Antibody responses against PfSE in P. falciparum (square) and P. vivax (diamond) exposed subjects. Each symbol represents the antibody titre of one individual. Dotted lines show cut-off values calculated from a mixture model as described in materials and methods. Solid lines show the median antibody titres in each group. (0.03 MB DOC) [file ppat.1000770.s001.doc]

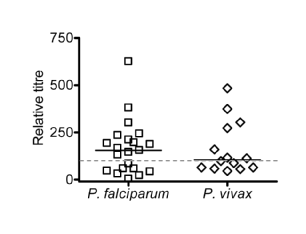


**Figure S1.** Antibody responses against PfSE in *P. falciparum* (*square*) and *P. vivax* (*diamond*) exposed subjects. Each symbol represents the antibody titre of one individual. Dotted lines show cut-off values calculated from a mixture model as described in materials and methods. Solid lines show the median antibody titres in each group.
